# High level of Sema3C is associated with glioma malignancy

**DOI:** 10.1186/s13000-015-0298-9

**Published:** 2015-06-02

**Authors:** Paulina Vaitkienė, Daina Skiriutė, Giedrius Steponaitis, Kęstutis Skauminas, Arimantas Tamašauskas, Arunas Kazlauskas

**Affiliations:** Laboratory of Neurooncology and Genetics, Neuroscience Institute, Lithuanian University of Health Sciences, Eiveniu str. 4, Kaunas, LT 50009 Lithuania

**Keywords:** Semaphorin, Sema3C, Glioma, Survival, Protein expression

## Abstract

**Background:**

Malignant gliomas are characterized by the tendency of cancerous glial cells to infiltrate into normal brain tissue, thereby complicating targeted treatment of this type of cancer. Recent studies suggested involvement of Sema3C (semaphorin 3C) protein in tumorigenesis and metastasis in a number of cancers. The role of Sema3C in gliomagenesis is currently unclear. In this study, we investigated how expression levels of Sema3C in post-operative glioma tumors are associated with the malignancy grade and the survival of the patient.

**Findings:**

Western blot analysis was used for detection of Sema3C protein levels in 84 different grade glioma samples: 12 grade I astrocytomas, 30 grade II astrocytomas, 17 grade III astrocytomas, and 25 grade IV astrocytomas (glioblastomas). Sema3C mRNA levels in gliomas were analysed by real-time PCR. Several statistical methods have been used to investigate associations between Sema3C protein and mRNA levels and clinical variables and survival outcome. The results demonstrated that protein levels of Sema3C were markedly increased in glioblastomas compared to grade I-III astrocytoma tissues and were significantly associated with the shorter overall survival of patients. High accumulation of Sema3C positively associated with the age of patients and pathological grade, but did not correlate with patient’s gender. Sema3C mRNA levels showed no association with either grade of glioma or patient survival.

**Conclusions:**

The data presented in this work suggest that the increased levels of Sema3C protein may be associated with the progression of glioma tumor and has a potential as a prognostic marker for outcome of glioma patients.

**Virtual slides:**

The virtual slide(s) for this article can be found here: http://www.diagnosticpathology.diagnomx.eu/vs/1564066714158642

**Electronic supplementary material:**

The online version of this article (doi:10.1186/s13000-015-0298-9) contains supplementary material, which is available to authorized users.

## Findings

### Introduction

Malignant gliomas have a high proliferation rate, increased angiogenesis and invasive growth. There is rarely a clear border between the tumor and the surrounding brain parenchyma. This imposes a problem when trying to perform a complete surgical resection of the tumor. As a consequence, recurrent neoplasms are established, usually within few months after surgery, in the proximity of the resection zone [1]. The development and invasiveness of gliomas are under strong influence by the local microenvironment: a plethora of factors (e.g., VEGF and bFGF) are secreted by tumor cells to affect the local tissue, whereas, in response, brain parenchymal cells secrete ligand molecules to stimulate glioma invasion and/or change the local microenvironment into a more permissive one for the tumor progression [2]. A group of autocrine regulatory factors, semaphorins and their receptors, neuropilins and plexins, were originally characterized as constituents of the complex regulatory system responsible for the guidance of axons during the development of the central nervous system. However, a growing body of evidence indicates that different semaphorins can either promote or inhibit tumor progression processes such as tumor angiogenesis, tumor metastasis and tumor cell survival [3]. Most of the class 3 semaphorins (i.e., semaphorins 3A, 3B, 3D, 3 F and 3G) were shown to inhibit cell migration and seem to be endowed with antitumor properties [4]. Meanwhile, semaphorin 3C (Sema3C) promotes tumor migration and was highly expressed in metastatic tumor cells [5]. There is evidence that Sema3C also promotes endothelial cell proliferation, migration, and tube formation *in vitro* and might be involved in regulation of angiogenesis [6]. Recent study on gastric cancer cells provided yet another evidence that Sema3C assists tumor progression [4].

Regarding Sema3C association with glioma, relatively high mRNA levels of Sema3C have been detected in a number of glioma cell lines and also in short-term glioma cultures derived from surgically removed tumor tissue [7]. The role of Sema3C expression in gliomas is currently unclear. In this study, we evaluated the Sema3C expression in different grade glioma tissues to test our hypothesis that Sema3C might be associated with the malignancy of this type of tumor and patient outcome.

## Materials and methods

### Patients and tissue samples

Were investigated post-operative samples obtained from 84 patients diagnosed with different malignancy grade gliomas: 12 grade I astrocytomas (pilocytic), 30 grade II astrocytomas (diffuse), 17 grade III astrocytomas (anaplastic), and 25 grade IV astrocytomas (glioblastomas). All glioma tumor samples were collected in Neurosurgery Clinics of Hospital (NCH) of Lithuanian University of Health Sciences (Kaunas, Lithuania) during the period from the year 2003 to 2012 with informed consent from patients. Tumor samples were collected, following written informed consent, in accordance with the Lithuanian regulations and the Helsinki Declaration. Written informed consent was obtained for every patient under the approval of the Ethics Committee, Lithuanian University of Health Sciences. Database closure was in September 2013. Diagnoses were established by pathologists at the NCH according to the World Health Organization (WHO) classification. Glioma samples were stored in liquid nitrogen before used. The following clinical data were collected for each patient: age at the time of the operation, gender, and patient status. The overall survival of the patient was calculated from the date of the operation to the date of death or the last recorded contact with the live patient. None of the patients had received chemotherapy or radiation before surgery.

### Whole-tissue extract preparation and Western blot analysis

Whole-tissue extracts of the tumor samples have been routinely prepared by resuspending the sample (100–200 μg) in RIPA lysis buffer (50 mM Tris–HCl, pH 7.5, 150 mM NaCl, 1 % Igepal CA-630 (Sigma-Aldrich), 0.5 % sodium deoxycolate, 0.1 % SDS) supplemented with a protease inhibitor cocktail (Sigma-Aldrich) and homogenizing using an ultrasonic sonifier (500-Watt Ultrasonic Processor, Cole-Parmer). Subsequently, the extracts were cleared by centrifugation for 30 min at 13.000 × g at 4 °C. 80 μg of the total extract protein were fractionated by 7.5 % SDS-PAGE and transferred to nitrocellulose membranes. Immobilized proteins were incubated for 2 h at 25 °C with the primary rabbit antibody against Sema3C (C-terminus, Antibodies-Online, catalog No. ABIN651266, dilution 1:500) in blocking solution (5 % nonfat milk in phosphate-buffered saline (PBS)). After extensive washing in PBS-T buffer (PBS supplemented with 0.5 % Tween-20), membranes were incubated with the horseradish peroxidase- (HRP-) conjugated anti-rabbit secondary antibody (Life Technologies, catalog No. 656120, dilution 1:2000) for 1 h at 25 °C. For detection of β-actin on the same membranes, the membranes were first cleared of the Sema3C antibody complexes by washing in the mild-striping buffer (25 mM glycine, 2 % SDS, pH 2.0) and reprobed with the primary monoclonal mouse antibody against β-actin (Antibodies-Online, catalog No. ABIN559692, dilution 1:2000) for 1 h at 25 °C followed by incubation with the HRP-conjugated anti-mouse secondary antibody (Life Technologies, catalog No. 626520, dilution 1:2000) for 1 h at 25 °C. Immunocomplexes were visualized using enhanced chemiluminescence reagents (Life Technologies) and documented by using gel imaging system GelDoc-It2 (Analytika Jena AG). Values of Sema3C and β-actin signals were calculated by using image analysis program ImageJ (National Institutes of Health, U.S.A.).

In the experimental setup, the whole set of 84 glioma tumor samples was organized in 6 groups each of which containing of 16–17 tissue extracts prepared from tumors of different malignancy grade (groups 1–4 contained samples of grades I-IV, whereas groups 5 and 6 contained only grades II-IV due to the scarcity of the grade I and grade III material). A separate immunoblot analysis was performed on a group of samples containing arbitrarily chosen representative samples from each of the 6 sample groups. Sema3C signals obtained from these group representatives were used to normalize Sema3C values (in addition to the normalization with the β-actin values) in all 6 membranes thereby enabling us to minimize diferences between separately processed membranes. Finally, the normalized Sema3C signal value of each tumor sample was divided by the average signal value calculated from the total of 84 samples resulting to the final value of the relative Sema3C protein expression.

### RNA extraction, cDNA synthesis and quantitative RT-PCR

Total RNA from cryogenically homogenized tumor tissue was purified using TRIzol Reagent (Ambion, Life Technologies). To increase the yield of RNA, homogenate was additionally sonicated using ultrasound (500-Watt ultrasonic processor, Cole Parmer). Reverse transcription (RT) was carried out using RevertAid H Minus M-MuLV Reverse Transcriptase (Thermofisher Scientific) and random hexamer primers (Thermofisher Scientific) in a total reaction volume of 20 μl according to the manufacturer’s protocol. For inhibition of mRNA degradation RiboLock RNase inhibitor (ThermoFisher Scientific) was used. After synthesis cDNA stock was stored at −80 °C. Sema3C mRNA expression was analyzed using quantitative real-time RT-PCR SYBR Green I in 3 replicates on 7500 Fast Real-time PCR detection system (Applied Biosystems) and Relative Quantitation method (ΔΔCT). Reactions have been assembled into a total volume of 12 μl, which included: 15 ng of the cDNA, 6 μl Maxima Hot Start PCR Master Mix (Thermofisher Scientific) with Hot start Taq DNA polymerase, primers for *SEMA3C* 5′-CAAAGATCCCACACACGGCT-3′(sense) and 5′-ACTTGGTCCTCTGATCTCCTCC-3′(antisense, amplicon length: 141 bp) and *ACTB* (encoding human β-actin) 5′-AGAGCTACGAGCTGCCTGAC-3′(sense) and 5′-AGCACTGTGTTGGCGTACAG-3′(antisense, amplicon length: 184 bp) to a total concentration of 0.5 μM and 0.1 μM, respectively, and nuclease-free water. PCR has been carried out for 40 cycles consisting of 95 °C for 30 s., 60 °C for 30 s., and 72 °C for 30 s. Fluorescent data were converted to threshold cycle (CT) measurements. ΔΔCT values were calculated from averaged replicates CT values according to the formula: ΔΔCT = (ΔCT_test smaple_ = CT *SEMA3C* – CT *ACTB*) – (ΔCT_reference sample_ = CT *SEMA3C* – CT *ACTB*). Differences between the plates were equalized by using the reference sample (human normal brain RNA sample, see below) Ct value realignment between experiments. To be able to quantify samples in 95 % of cases, samples with standard deviation more than 0.25 (Ct between replicates) were eliminated from analysis. The final result was given as Log_2_ of 2^-(ΔΔCT)^ calculation. Human normal brain RNA sample “FirstChoice Human Brain Reference RNA” (Ambion, cat. No. AM6050), which was a pool of RNAs assembled from multiple donors from several brain regions, as described by the manufacturer, served as a control sample for standard curve design. Standard curve parameters were as follows: for *SEMA3C*: efficiency 100.2 %, R^2^ 0.99, slope −3.31; for *ACTB*: efficiency 101.2 %, R2 0.997, slope −3.29, thereby confirming the suitability of PCR conditions and primers for mRNA quantitation (also please see the Additional file 1: Figure S1).

### Statistical analysis

SPSS Statistics 19 (SPSS Inc., Chicago, IL) software package was used for statistical analysis. Differences in Sema3C protein and mRNA expression between different grade groups were evaluated using the Kruskal-Wallis test. Linear correlation between Sema3C protein and mRNA expression was examined using Spearman’s Correlation Coefficient analysis. Associations between Sema3C protein level and clinical features of glioma patients were analyzed by Chi-Square Test. The Kaplan-Meier method was used to estimate survival functions. For comparing survival time distribution between groups the log-rank test was used. Cox regression model was utilized to perform a multivariate analysis. The level of significance was set to p <0.05. False Discovery Rate (FDR) analysis was carried out by using SPSS script designed to “Perform the Benjamini and Hochberg FDR procedure”. Analysis showed that the Sema3C protein expression results are still statistically significant with the FDR criterion of 0.05.

### Ethical standards

Experiments described in the manuscript comply with the current laws of the country (Republic of Lithuania) in which they were performed.

## Results

### Analyses of Sema3C expression in different grade gliomas

**Fig. 1:**
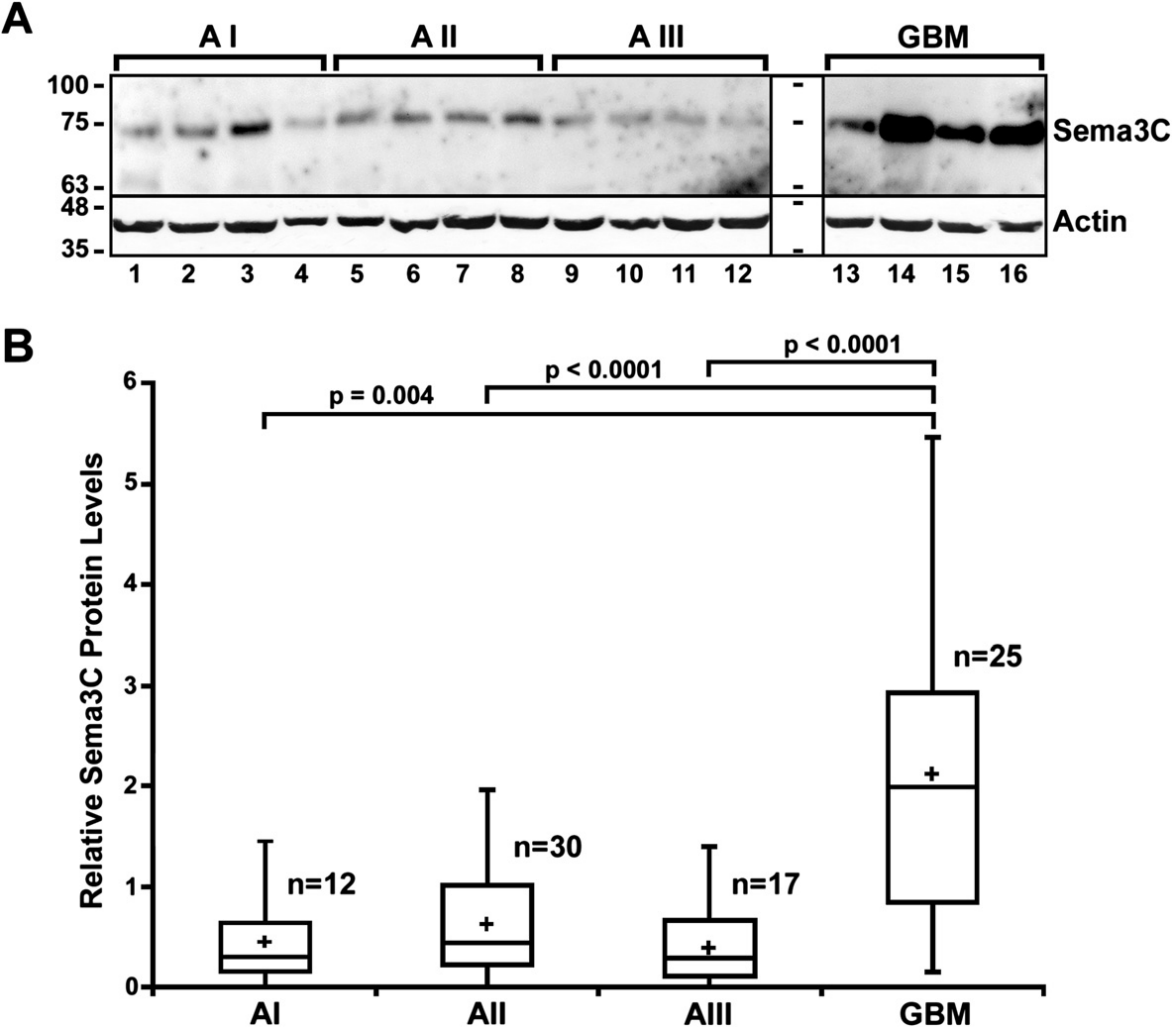
Protein levels of Sema3C are upregulated in glioblastoma. **a** Representative example of a Western blot showing Sema3C protein expression in glioma tumor samples of differnet maligancy grades. Astrocytomas of WHO grade I, II, III, and IV (glioblastoma) are indicated as *AI*, *AII*, *AIII*, and *GBM*, respectively. Positions of Sema3C *(Sema3C),* β-actin *(Actin)* and kDa values of the protein size marker (one before *lane 1* and the second between *lanes 12* and *13*) are indicated. **b** Box plots of relative expression measurements of Sema3C obtained by Western blot analysis of astrocytoma samples. The line inside each box represents the median, the plus symbol (+) inside the box represent the mean, and the lower and upper edges of the boxes represent the 25th and 75th percentiles, respectively, and upper and lower lines outside the boxes represent minimum and maximum values (error bars). The Kruskal-Wallis test shows that there are statistically significant differences of relative Sema3C expression when glioblastomas are compared to grade I, II and III astrocytomas (p = 0.004, p < 0.0001, and p < 0.0001, respectively)

By using immunoblot analysis (Western blot), we aimed to test whether expression of Sema3C at the protein level is associated with the different histopathological grades of glioma. For this purpose, protein extracts (whole-tissue extracts) were prepared from 84 tumor samples of glioma patients: 12 samples of grade I, 30 samples of grade II, 17 samples of grade III, and 25 samples of grade IV (glioblastoma) tumors. Relative amounts of Sema3C and β-actin (internal control) protein level in each of the extracts was determined by using Sema3C- and β-actin-specific antibodies (Fig. 1a, also please see the Additional file 1: Figure S2 for Sema3C protein levels in the entire set of glioma samples). The analysis of the obtained data demonstrated that levels of Sema3C protein in glioblastoma (grade IV) were significantly higher than those of the lower grade (I–III) glioma tissues (p < 0.0001, Fig. 1b). Differences in Sema3C protein expression were not significant between grade I, II and III gliomas according to the Kruskal-Wallis test (p > 0.05).

**Fig. 2:**
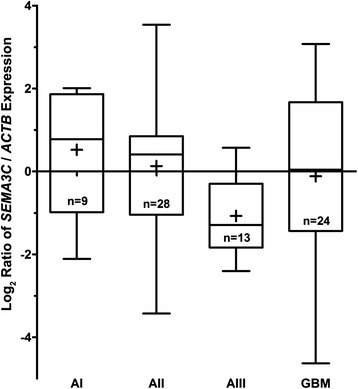
Relative *SEMA3C* mRNA expression in different malignancy grade astrocytic gliomas. The markings of malignancy grade are the same as in Fig. 1. Real-time PCR data shown as log_2_ of fold change of SEMA3C/*ACTB* mRNA expression. The line inside box represent median, the plus symbol (+) inside the box represent the mean, and the lower and upper edges of the boxes represent 25th an 75th percentile, respectively. Upper and lower lines outside the box represent minimum and maximum values (error bars)

Next, we decided to examine whether there is a relationship between mRNA levels of Sema3C and malignancy grade of the glioma tumor. For this purpose, we analyzed Sema3C mRNA levels by using quantitative real-time PCR (RT-PCR) technique on mRNA isolated from the same set of glioma samples tha has been used for Western blot studies except for 10 samples, which were not included because of the scarcity of the material or due to the exceed value of standard deviation of CT. Sema3C mRNA values determined in 74 gliomas (9 grade I astrocytomas, 28 grade II astrocytomas, 13 grade III astrocytomas, and 24 grade IV astrocytomas) were normalized to mRNA levels of the β-actin-encoding gene *ACTB*, which has been used as an internal control. Contrary to our expectations, Sema3C mRNA levels, evaluated by using the Kruskal-Wallis test, revealed no significant association with the tumor grade (Fig. 2), and there was no linear relationship found between Sema3C mRNA and protein levels (Spearman’s r = 0.056, p > 0.05).

### Correlation of Sema3C protein level with clinicopathological characteristics and patient survival

To determine the significance of the increased Sema3C protein expression in glioma, we analyzed the relationship between Sema3C protein levels and the clinicopathological features of glioma patients. Sema3C protein level values obtained by Western blot from the the complete set of 84 different grade glioma samples were ranked into three categories: values that were lower than or equal to the 25th percentile were ranked as “low” Sema3C protein level, values falling between the 25th and 75th percentiles were considered as “medium” Sema3C protein level, and values that were higher than or equal to the 75th percentile were ranked as “high” Sema3C protein level. “Low” protein levels of Sema3C were determined in 22 (26.2 %), “medium” Sema3C levels were determined in 41 (48.8 %), and “high” Sema3C levels were determined in 21 (25.0 %) glioma tumors. As shown in Table 1, we found positive significant relationship between Sema3C protein level and high tumor malignancy (p < 0.0001). This result suggests that accumulation of high levels of Sema3C was positively associated with the progression of glioma. Sema3C levels also positively associated with the age of patient (p < 0.0001), but did not correlate with gender (p >0.05, Table 1).

**Table 1:** Association of Sema3C protein level in human glioma tissues with different clinicopathological features

Variable	Number of cases	Sema3C protein level			p-value
		Low n (%)	Medium n (%)	High n (%)	
Overall	84	22 (26.2)	41 (48.8)	21 (25.0)	
Age (Years)					
≤50	49	19 (38.8)	27 (55.1)	3 (6.1)	0.0001
>50	35	3 (8.6)	14 (40.0)	18(51.4)	
Gender					
Male	38	12 (31.6)	19 (50.0)	7 (18.4)	0.37
Female	46	10 (21.8)	22 (47.8)	14 (30.4)	
Pathological grade					
grade I	12	5 (41.7)	7 (58.3)	0 (0.0)	0.0001
grade II	30	8 (26.7)	18 (60.0)	4 (13.3)	
grade III	17	8 (47.0)	8 (47.0)	1 (6.0)	
grade IV	25	1 (20.0)	8 (16.0)	16 (64.0)	

**Fig. 3:**
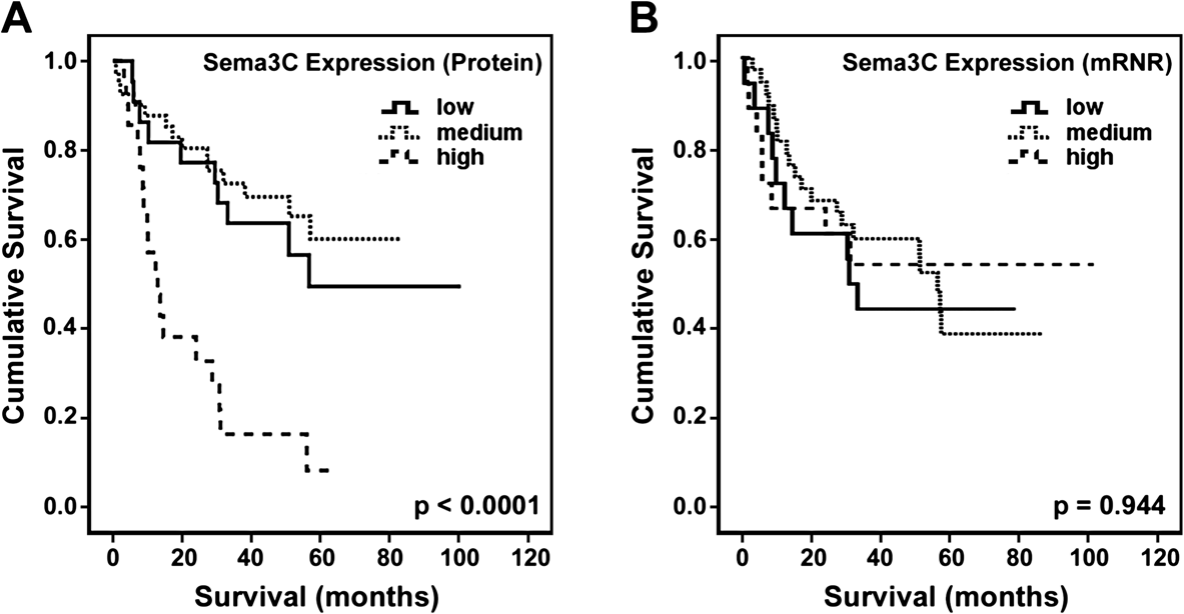
Kaplan-Meier survival curves representing the overall survival of glioma patients. The whole set of glioma samples stratified by relative protein or mRNA levels to “low”, “medium”, and “high” level categories, as described in the text above. **a** The relationship between the protein level of Sema3C and patient survival. N = 84, log-rank test, *χ*2 = 29.931; df = 2, p < 0.0001. **b** The relationship between the mRNA level of Sema3C and patient survival. N = 74, log-rank test, *χ*2 = 0.115; df = 2, p = 0.944

The Kaplan–Meier analysis using the log-rank test was performed to determine the association of Sema3C expression with clinical outcome of glioma patients (Fig. 3a). The results showed that high levels of Sema3C protein in glioma samples was markedly associated with a shorter overall survival (p < 0.0001). Similarly, the Univariate Cox regression analysis (Table 2) also indicated that clinical variables including age (p < 0.0001), pathological grade (I-II vs. III-IV WHO grade gliomas; p < 0.0001), and Sema3C protein levels (p = 0.0001) were significantly associated with the overall survival. However, the Multivariate Cox regression analysis by using enter method failed to demonstrate the level of Sema3C protein as an independent predictor for overall survival of glioma patients (p > 0.05) suggesting that, in addition to the Sema3C protein levels, the combination with other two clinical factors, pathological grade (p < 0.0001) and age (p < 0.0001), is critical for making predictions of the overall survival in glioma.

**Table 2:** Cox regression analysis for glioma patients (n = 84)

Variable	Univariate			Multivariate		
	HR	95 % CI	p-value	HR	95 % CI	p-value
Age (years)	1.062	1.045-1.080	<0.0001	1.064	1.034-1.095	<0.0001
Gender (female vs. male)	0.904	0.582-1.404	0.654			
WHO grade (I-II vs. III-IV)	4.879	2.892-8.231	<0.0001	4.081	1.918-8.682	<0.0001
SEMA3C protein level						
low (reference)	-	-	-	-	-	-
medium	0.756	0.336-1.703	0.500	0.545	0.234-1.268	0.159
high	3.613	1.638-7.969	0.001	0.826	0.313-2.183	0.700

We also examined how Sema3C mRNA levels reflect upon the survival of glioma patient. The method expression level ranking (“low”, “medium”, and “high”) used for Sema3C protein has also been applied to mRNA values. In contrast to Sema3C protein data, no significant association between Sema3C mRNA level and patient overall survival has been observed (Figure 3b, log-rank test p > 0.05).

## Discussion

Glioma is one of the most aggressive human tumors, the prognosis of which remains extremely poor despite multimodal treatment by surgery, radiotherapy and chemotherapy. Malignant glioma tumors are characterized by their propensity towards extensive invasion into surrounding healthy tissue and resistance to both chemo- and radiation therapy. There is increasing interest in identification of prognostic molecular biomarkers, which could allow to better capture the status of tumor, provide knowledge about glioma formation and progression and could be the potential drug targets. The process of tumor cell invasion is a complex combination of cellular and molecular events involving the breakdown of extracellular matrix (ECM) components, cell detachment, and migration through the basement membrane and stroma [8]. Semaphorins were initially characterized as regulators of cell migration that play role in repulsive axon guidance but are now recognized as key players in morphogenesis and homeostasis over a wide range of organ systems [9]. A growing body of evidence indicates that various semaphorins can modulate tumor progression through either promotion or inhibition of processes such as tumor angiogenesis, tumor metastasis and cancer cell survival [3]. Semaphorin 3C is a member of the class 3 family of secreted semaphorins that are known to play important roles in regulating neuronal as well as vascular patterning [10], mesenchymal cell differentiation, and neural crest cell migration [11]. In addition, Sema3C is a positive regulator of endothelial cell functioning sucha as cell survival, proliferation, adhesion, migration, and tube formation *in vitro* by stimulating integrins and VEGF secretion [6]. Taking into account this functional background of Sema3C, perhaps it is not surprising to witness an increasing number of studies showing that aberrant expression levels of this protein is associated with a number of cancer phenotypes. It has been shown, for example, that Sema3C is highly expressed in several human cancer types. The expression of Sema3C was found to be upregulated in metastatic cells of lung adenocarcinoma [12]. High Sema3C expression increased motility and invasion of prostate cancer cells [13], whereas in ovarian cancer high levels of Sema3C were associated with shorter patient survival [14]. A very recent study has demonstrated that Sema3C signaling could play a role as a central regulator of glioma stem cell survival and glioblastoma progression via activation of the Rac1/NF-κB signaling pathway [15].

With regard to Sema3C expression in gliomas, one of the prominent studies on this topic reported high Sema3C mRNA expression in the majority of the tested glioma cell lines and primary glioma cell cultures derived from surgically removed tumor tissue [7]. Experiments conducted by Man and colleagues showed strong Sema3C staining compared to normal human brain tissue in a subpopulation of glioblastoma cells [15]. In the same laboratory, a microarray anaylsis of glioblastoma tissues revealed that Sema3C was overexpressed in a subpopulation of tumor cells in 30 out of 35 (85.7 %) cases but was barely detectable in normal brain tissue [15]. Our study for the first time examined Sema3C expression at protein levels in astrocytoma tissues of different malignancy grade. We observed a marked increase of Sema3C in glioblastomas compared to lower grade (i.e., WHO grades I, II, and III) astrocytomas (Fig. 1) indicating that the aberrant expression of Sema3C may be strongly associated with the advancement of glioma malignancy. Sema3C upregulation was distinctly associated with the poor prognosis in glioma patients, especially with high WHO grade (Fig. 3a). However, the real-time PCR analysis of the same set of glioma samples showed no association between Sema3C mRNA levels and malignancy grade of the tumor (Fig. 2) as well as patient survival (Fig. 3b). This result, although different from the observations mentioned earlier in this section [7, 15], should not be seen as a surprise because the issue regarding the expression of Sema3C at mRNA levels in gliomas has not been resoved yet. For example, Laffaire and colleagues by using genomic methylation and comparative gene expression profiles found that *SEMA3C* gene is differentially methylated in both low-grade gliomas and glioblastomas compared with normal brain controls. *SEMA3C* methylation frequency has been determined in 72 % of low-grade gliomas and in 56 % of glioblastomas [16], whereas the gene expression data placed *SEMA3C* among genes that were prominently downregulated in gliomas. However, the validation of experimental data on *SEMA3C* with the TCGA (The Cancer Genome Atlas) glioblastoma data set revealed different pattern of expression and methylation of *SEMA3C*, thus, leaving the question regarding *SEMA3C* expression in gliomas open [16]. In any case, we would like to stress here that we do not rule out the involvement of the transcriptional regulation of the *SEMA3C* gene in the process of gliomagenesis since, given the heterogeneuos composition of glioma tumor (especially high grade), it is plausible that the active *SEMA3C* transcription might be going on in a specific subpopulation of cells, such as glioma stem cells [15]. Expression of Sema3C in those cells has been demonstrated to be important for growth and invasion of the glioma tumor [15].

While searching for alternative explanations of the increase of Sema3C levels in high grade gliomas, we would like to point out that the most predominant 70 kDa Sema3C isoform in our immunoblots most likely represents the cleavage product of Sema3C generated by one of the proteases (e.g., furin-like pro-protein convertase (FPPC) [17] since the full-length Sema3C protein has been reported of 95 kDa in size, which was detected in the human breast cancer cell line MCF7 [5]. Therefore, we suggest the possibility that the high accumulation of Sema3C in glioblastomas may be the result of: i) lower activity of certain Sema3C-targetting protease(s) compared to lower grade astrocytomas or ii) the higher degree of protection of Sema3C against ECM proteases while being in a complex with cell surface receptors (neuropilins, plexins [3, 15]) in glioblastomas. The notion that Sema3C processing may differ between glioma grades is at some extent supported by our observation that a short, approximately 45 kDa fragment, which was recognized by Sema3C antibody in immunoblots, appeared more often in grade I-III astrocytoma samples rather than in glioblastomas (Additional file 1: Figure S3). The importance of the site-specific processing of secreated semaphorins for their function has been demonstrated in a number of studies. For example, Sema3 proteins possess from one to three furin consensus sequences (RXRR), the cleavage at which, in cases of Sema3A and Sema3F, are critical for binding to neuropilin-1 and inducing antiangiogenic effects on endothelial cells [18, 17]. The cleavage of the short basic domain at the C-terminal portion of Sema3C by metalloproteinase ADAMTS1 seem to be important for Sema3C-dependent induction of endothelial cell migration, whereas Sema3C processing to a short p65 isoform by FPPC renders it inactive [5]. Moreover, the FPPC cleavage-resistant Sema3C has been shown to inhibit proliferation and migration of lymphatic endothelial cells and human umbilical vein endothelial cells [19]. We do not know whether the 70 kDa isoform of Sema3C detected in our immunoblots corresponds to the inactive p65 protein or, on the contrary, represents a hitherto uncharacterized biologically active form of Sema3C. This particular and other important questions regarding role Sema3C in gliomagenesis should be addressed in the future studies.

## Conclusion

Findings presented in our study show that the increased protein level of Sema3C is associated with the progression of glioma malignancy and poor patient survival. Further investigation will be required to evaluate Sema3C value as a molecular marker of glioma prognoses.

## Additional file

Additional file 1:
**This additional file includes supplementary figures S1-S3.**
