# Supplementary material for: High level of Sema3C is associated with glioma malignancy
Source: Diagn Pathol. 2015 Jun 2;10:58. doi: 10.1186/s13000-015-0298-9 (PMC4450827; doi:10.1186/s13000-015-0298-9)
Supplement: Additional file 1: — This additional file includes supplementary figures S1-S3. [file 13000_2015_298_MOESM1_ESM.pdf]

Figure S1

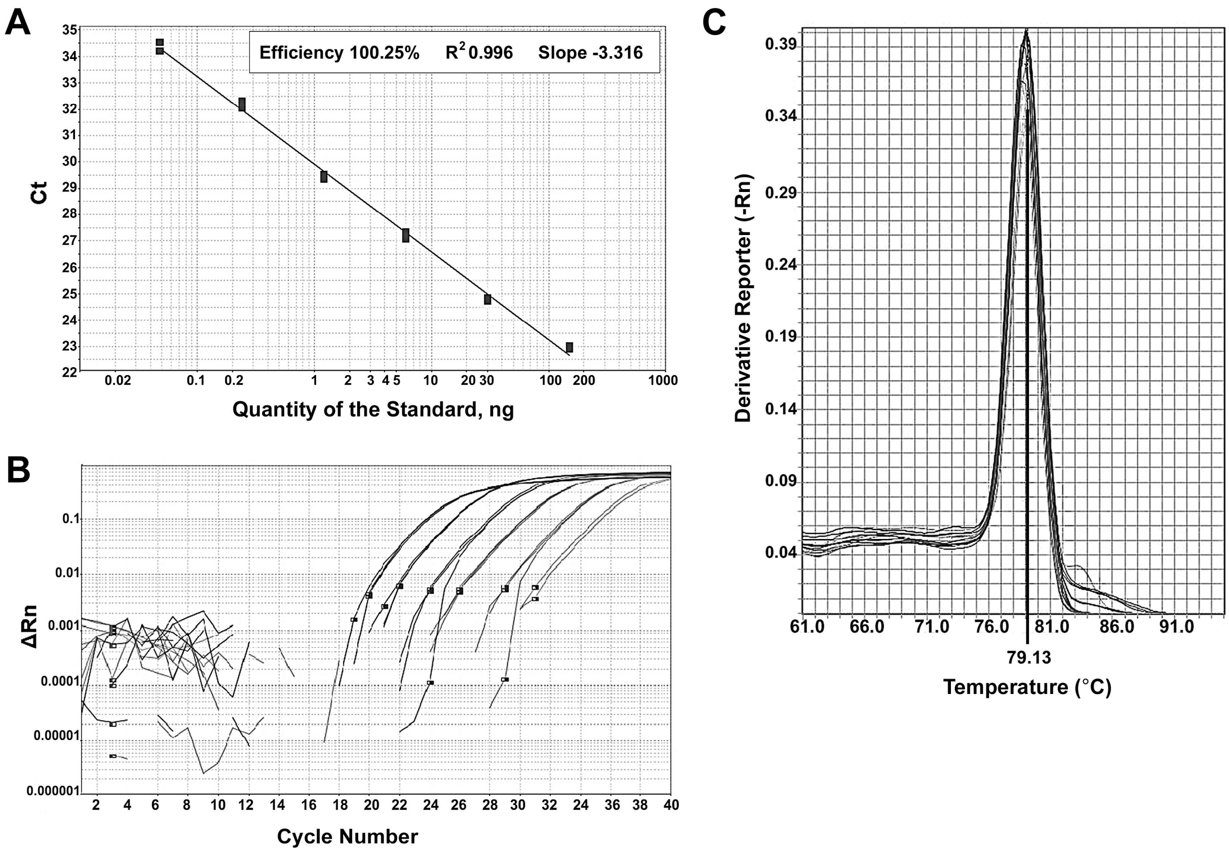

Optimization of Real-Time PCR conditions. (A) SEMA3C standard curve consisted of 6 points of the reference sample (reference human brain RNA), 5x dilutions (150, 30, 6, 1.2, 0.24, 0.048 ng/well). All dilution points were done in 3 replicates. SEMA3C standard curve parameters: efficiency 100.2 %, R2 0.99, slope -3.31, thereby confirming the suitability of PCR conditions and primers for mRNA quantitation. (B) Amplification plot. (C) Melting curve analysis of PCR products.

Figure S2

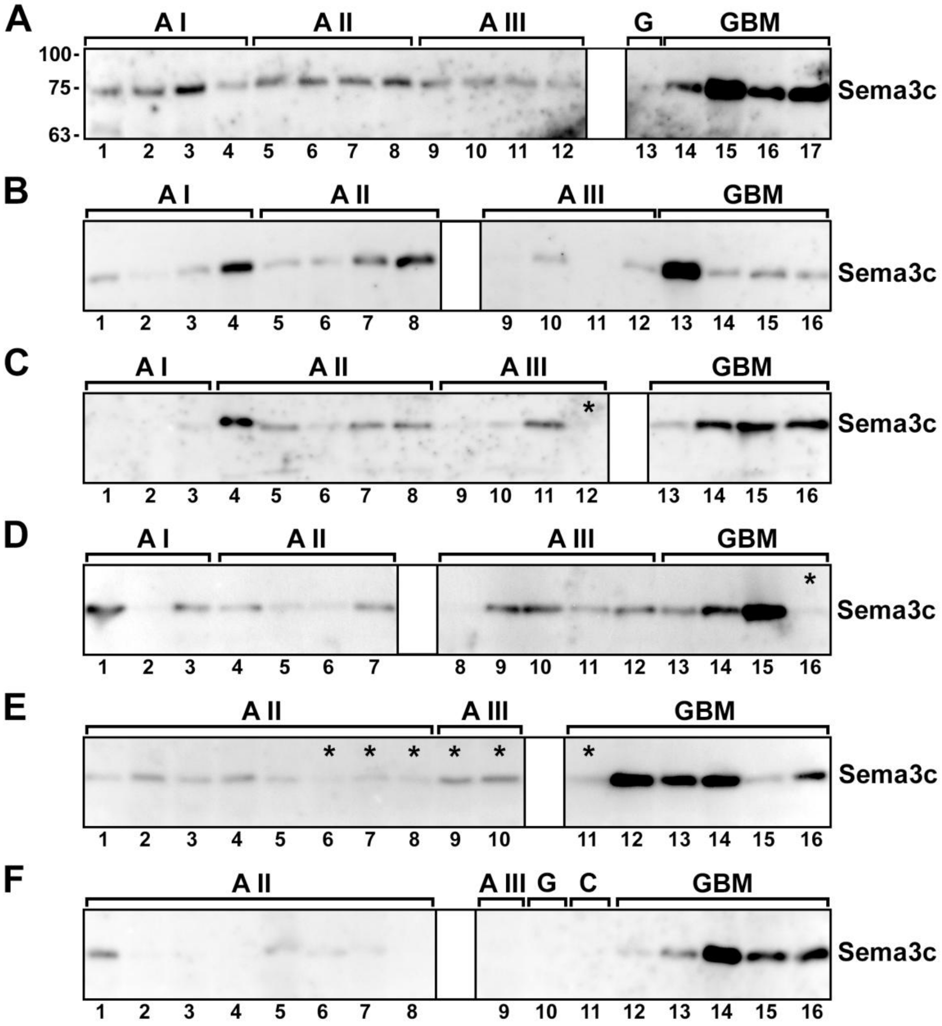

Western blots showing Sema3C protein levels in different grade glioma tumors. Astrocytomas of WHO grade I, II, III, and IV (glioblastoma) are indicated as *AI*, *AII*, *AIII*, and *GBM*, respectively. Positions of Sema3C *(Sema3C)* and kDa values of the protein size marker (panel *A*) are indicated. For comparison, we used a postoperative sample of the gliosis, which has been surrounding cavernoma (indicated as *G*), and the commercial lysate prepared from normal cortex (indicated as *C*). Samples that were not included in alyses due to various technical reasons (duplicates, lack of actin data, etc.) are marked with *asterisk*.

Figure S3

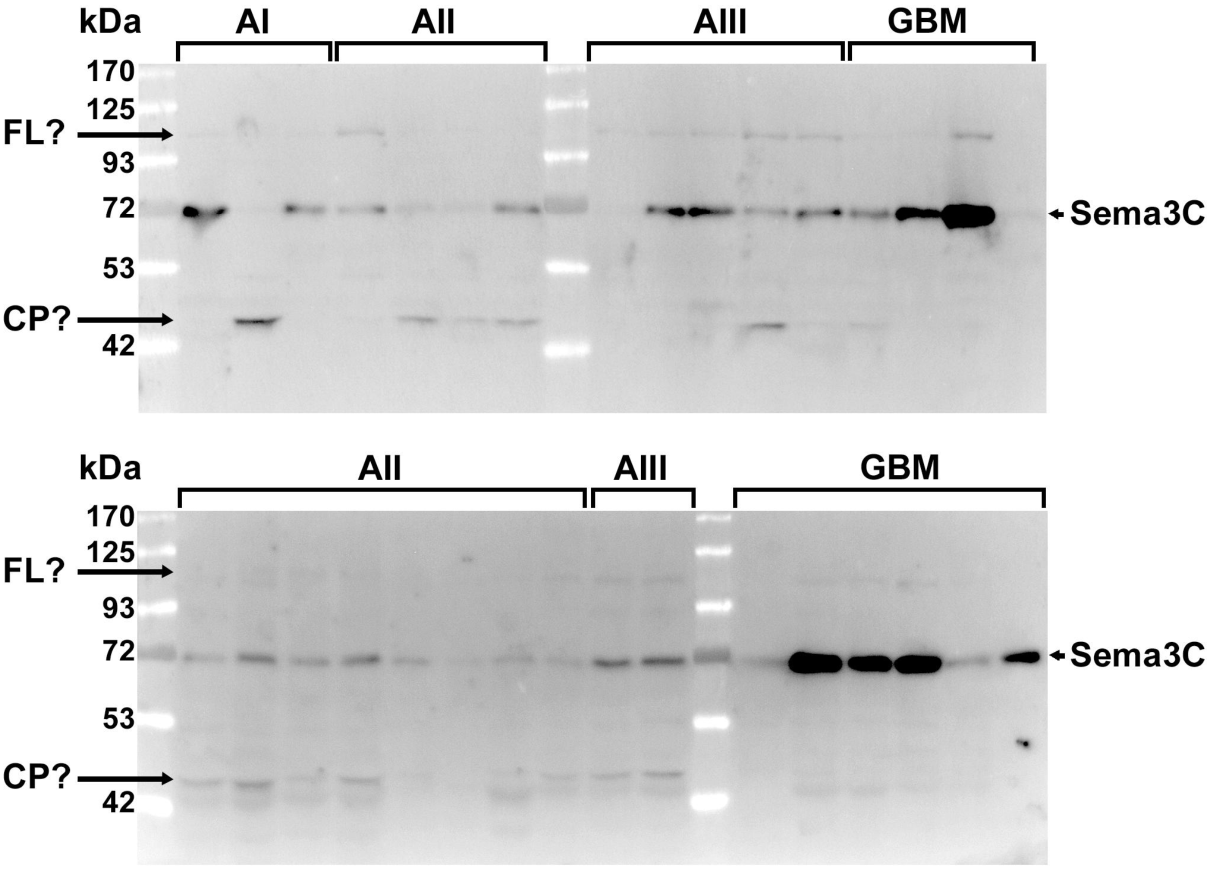

The two full-sized Western blot images are shown as an example of variation of Sema3C isoform patterning in different grade gliomas. The most prominent is the 70 kDa Sema3C protein band, the intensity of which has been used for measuring Sema3C protein level in individual glioma samples (Sema3C). A higher migrating band (FL), probably, is the full-length isoform of Sema3C. The band of approximately 45 kDa might be a putative cleavege fragment (CP) of Sema3C generated, as we speculate, by one of the ECM proteases (e.g., furin-like protease). Astrocytomas of WHO grade I, II, III, and IV (glioblastoma) are indicated as AI, AII, AIII, and GBM, respectively. The kDa values of the protein size marker bands are indicated.
